# Dracunculiasis Eradication: Are We There Yet?

**DOI:** 10.4269/ajtmh.18-0204

**Published:** 2018-06-04

**Authors:** Donald R. Hopkins, Ernesto Ruiz-Tiben, Mark L. Eberhard, Adam Weiss, P. Craig Withers, Sharon L. Roy, Dean G. Sienko

**Affiliations:** 1 The Carter Center, Atlanta, Georgia;; 2 Centers for Disease Control and Prevention, Atlanta, Georgia

## Abstract

This report summarizes the status of the global Dracunculiasis Eradication Program as of the end of 2017. Dracunculiasis (guinea worm disease) has been eliminated from 19 of 21 countries where it was endemic in 1986, when an estimated 3.5 million cases occurred worldwide. Only Chad and Ethiopia reported cases in humans, 15 each, in 2017. Infections of animals, mostly domestic dogs, with *Dracunculus medinensis* were reported in those two countries and also in Mali. Insecurity and infections in animals are the two main obstacles remaining to interrupting dracunculiasis transmission completely.

## INTRODUCTION

The global Dracunculiasis Eradication Program (DEP) is much closer to its goal of stopping transmission of dracunculiasis (guinea worm disease) since the previous review in this series was published 5 years ago.^1^ This article describes the status of the program as of the end of 2017.

We described in previous reports the parasite and the strategies and interventions being used to eradicate it.^1,2^ Dracunculiasis (guinea worm disease) is caused by the nematode parasite *Dracunculus medinensis*, and it is transmitted to humans in contaminated drinking water containing copepods (water fleas) that harbor infective larvae of the parasite. Recently, the potential infection of humans by eating poorly cooked or cured aquatic animals that have ingested infected copepods has emerged as a possible mode of infection.^3^ The larvae are expelled into water by adult female worms, and then, after human consumption of copepods or aquatic animals with infective larvae, the adult worms emerge through the skin of infected persons about 1 year after infection. Once the end of the worm has emerged through the skin, the remainder of the worm, up to 1 m long, must be removed. Emergence and removal of the worm is slow, painful, and often disabling due to secondary bacterial infections. Although usually not fatal, the disease has a serious adverse socioeconomic impact on the health, agricultural productivity, and school attendance of affected populations. Without medical care, persons are incapacitated for periods averaging 3 months. In the past, more than one-half of a village’s population might have been affected simultaneously during the main harvest or planting season. Until significant numbers of infections with *D. medinensis* were discovered in dogs in Chad in 2012, humans were the only known reservoir of infection. Individual infections last only 1 year, but people do not develop immunity to the parasite. There is no effective treatment or vaccine; however, the infection may be prevented by 1) educating villagers about the origin of the disease, 2) preventing infected persons and animals from entering sources of drinking water, 3) filtering all drinking water through a finely woven cloth that removes the copepods, 4) applying Abate larvicide (temphos; BASF Corp., Florham Park, NJ) to kill the copepods in ponds or other stagnant sources of drinking water, and 5) providing clean drinking water from safe sources, such as protected hand-dug or borehole wells.

The global eradication campaign began at the Centers for Disease Control and Prevention (CDC) in 1980. It was adopted as a subgoal of the International Drinking Water Supply and Sanitation Decade (1981–1990), and it has been led since 1986 by The Carter Center, which is at the head of a coalition that includes the ministries of health of the endemic countries, the CDC, the World Health Organization (WHO), and the United Nations Children’s Fund as major partners and thousands of village volunteers and supervisory health staff. The coalition is supported by numerous donor agencies, governments, foundations, and other institutions. When The Carter Center began leading the global campaign after the CDC, there were an estimated 3.5 million cases of dracunculiasis worldwide.^4^ At the World Health Assembly (WHA) in 2004, ministers of health set a target to stop transmission of dracunculiasis by the end of 2009.^5^ When that target date was not met, partly because of the ongoing civil war in Sudan and unexpected outbreaks in Chad, Ethiopia, and Mali, the global initiative resolved to interrupt transmission as soon as possible.

## CURRENT STATUS OF THE CAMPAIGN

**Figure 1.**
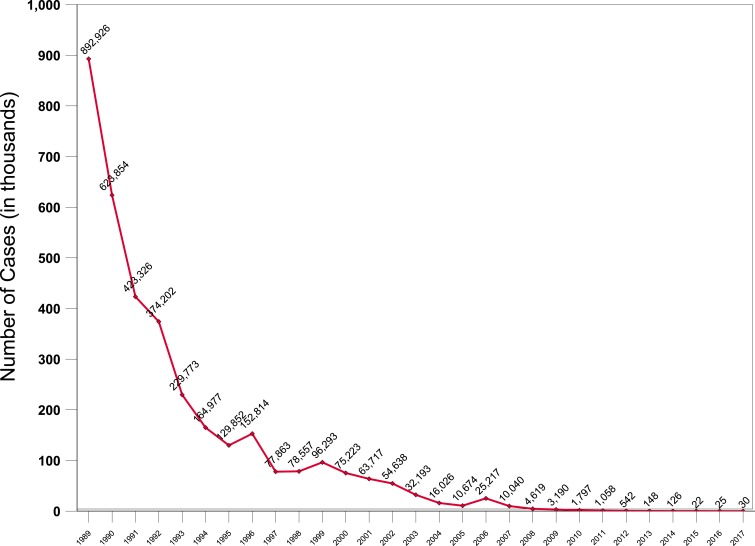
Number of reported cases of guinea worm disease in humans by year: 1989–2017. This figure appears in color at www.ajtmh.org.

**Figure 2.**
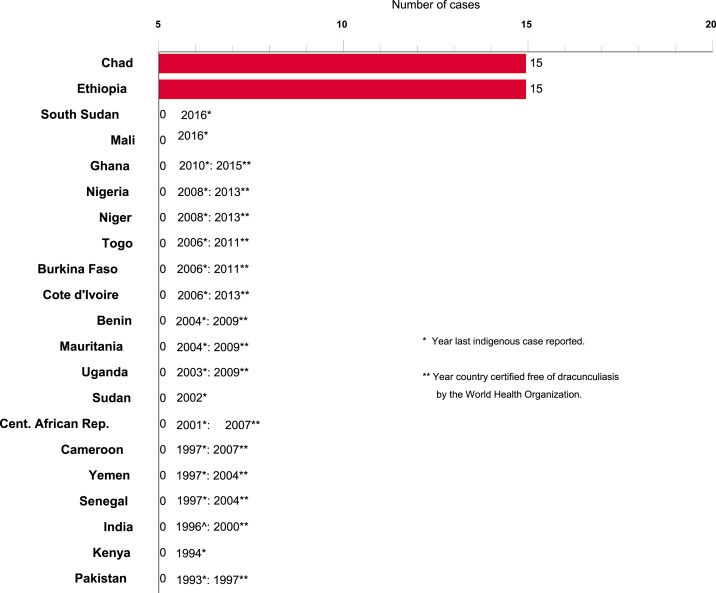
Distribution of 30 indigenous cases of dracunculiasis in humans reported during 2017. This figure appears in color at www.ajtmh.org.

As of the end of 2017, only 30 cases of dracunculiasis in humans were reported worldwide (down from 542 cases in 2012), in 18 villages (down from 103 villages with indigenous cases in 2012), and only two countries reported cases in humans (down from four countries in 2012) (Figures 1 and 2). A third country reported infections only in animals. Mali and South Sudan each reported their last known cases in humans in 2015 and 2016, respectively. Chad and Ethiopia reported 15 cases each in 2017, whereas Chad, Ethiopia, and Mali reported 830, 15, and 10 infected animals, mostly domestic dogs, respectively. South Sudan reported only one infected domestic dog, in 2015. The Centers for Disease Control and Prevention has confirmed worms from the infected animals as *D. medinensis* by microscopic and/or molecular examination.^3^ The number of cases exported from one country to another has fallen from a peak of 154 cases in 2002 to three cases in 2012 and zero cases in 2013–2017.

### Chad.

**Figure 3.**
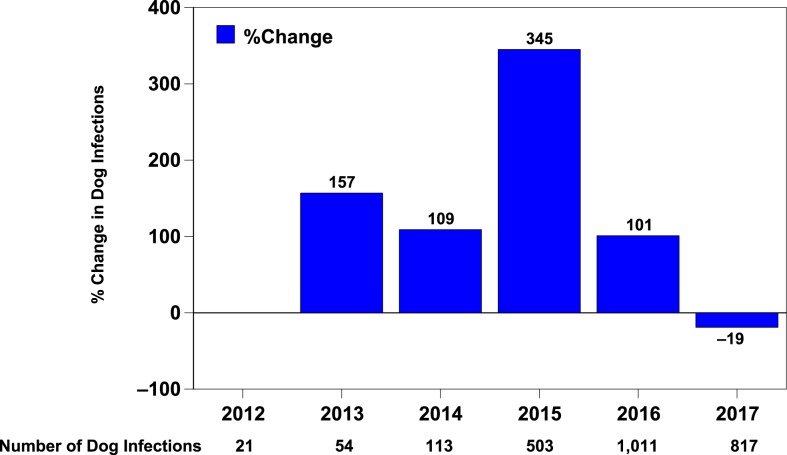
Chad Guinea Worm Eradication Program, dog guinea worm infections, and percent change from preceding year: 2012–2017. This figure appears in color at www.ajtmh.org.

**Figure 4.**
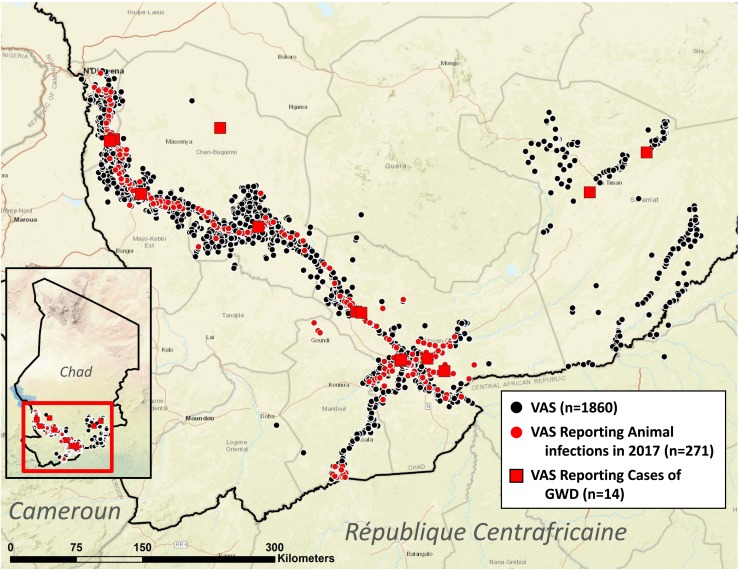
Villages under active surveillance (VAS), reporting animal infections with guinea worms, and cases of guinea worm disease (GWD) during 2017 (in color).

Cases of dracunculiasis were rediscovered in Chad in 2010 after the country had reported no cases during the previous decade. Chad has reported a total of 97 cases (representing 31 ethnicities in 78 villages) in 2010–2017, ranging from 9 to 16 cases per year (15 cases in 14 villages in 2017), and was declared endemic again in 2012, after three consecutive years of indigenous cases. The specific source locations of infection of the cases in Chad are unknown. The number of infected domestic dogs reported increased steadily from 27 in 2012 to 1,011 in 2016 before being reduced by 19% to 817 infected dogs in 271 villages in 2017, when the number of guinea worms removed from dogs fell by 31%, from 2,019 in 2016 to 1,386 (Figure 3). These were the first reductions in infected dogs and emerging guinea worms in Chad since infections in dogs were reported in 2012. Chad also reported a total of 33 infected cats in 2013–2017, including one wild cat in 2014. In 2014, Chad established a new category to denote its affected villages of concern for operational purposes: “1+ case village,” a village with one or more indigenous and/or imported guinea worm infection in a human, dog and/or cat in the current and/or previous calendar year. Most cases in humans peak during the rainy season in June–September, with no marked preponderance in adults versus children or males versus females. Dog infections peak in April–August. The vast majority of infections in humans and dogs occur in villages along the Chari River (Figure 4).

Although the guinea worms found in humans and in dogs in Chad are indistinguishable in the laboratory, the current outbreak has exhibited a “peculiar” epidemiology since it began, with unusually high numbers of infected dogs, few sporadic infections in humans, almost no clusters of cases in families or in villages in successive years, and no large outbreaks of human cases. Evidence to date suggests that transmission of the infection in Chad may involve a paratenic or transport host (intermediate hosts in which the parasite remains viable but does not develop) such as fish, frogs and/or another aquatic animal, and that humans and dogs may be infected by eating such aquatic animals raw or inadequately cooked or cured.^6^ Casual disposal of entrails during the mass harvesting of fish at the end of the dry season is believed to contribute to the possible alternative mode of transmission among dogs.^3^

The WHO assisted Chad after transmission was believed to have been interrupted in 2000, but Chad did not sustain its eradication program or surveillance for dracunculiasis. The Carter Center resumed assistance to Chad in 2011 at the request of the Ministry of Public Health, helping to reestablish active surveillance starting in 2012. At the end of 2017 Chad had 1,860 villages under active surveillance, of which 463 were “1+ villages.” It has offered a cash reward equivalent to US$100 for reporting a case of the disease for over a decade and began offering a reward equivalent to US$20 for reporting and tethering (containing) an infected dog in January 2015. In 2017, awareness of the cash reward for reporting a case in humans was estimated at 55% of persons queried and about 41% were aware of the reward for reporting an infected dog.

Chad’s Guinea Worm Eradication Program (GWEP) began implementing enhanced health education in 2013, urging villagers to cook their fish well and bury fish entrails. Tethering and daily monitoring of infected dogs and application of Abate in cordoned areas of the large lagoons (e.g., 2.5 × 2.2 km) along the banks of the Chari River in response to contamination events began in 2014. Domestic dogs in Chad are associated with specific households, which provide them some of the family’s food, sometimes drinking water, and access to raw fish entrails. The dogs accompany household members while farming, hunting, visits to market, etc. and also forage for food and water. As the river recedes during the dry season, smaller ponds appear in villages. These ponds are used for brick-making and gardening. They often contain fish and frogs, and are also accessible to dogs. More intense Abate application to ponds in communities with multiple dog infections began in 2017, when it was applied in 70 villages that contained 40% of the infected dogs in 2017. The rate of containment of infected dogs rose from 40% in 2014 to 68%, 66% and 76% in subsequent years, whereas the proportion of sampled households that were found to practice safe disposal of fish guts was 88% in 2016 and 83% in 2017. Fully 72% of 1+ villages had at least one source of safe drinking water in 2017. We believe the reduction in infected dogs and emerging guinea worms in 2017 probably resulted from the impact of tethering infected dogs, burying fish guts, and use of Abate. Chad’s National Assembly convened in June 2017 for a special briefing on the eradication program. The minister of public health launched an intensive nationwide communication campaign in July 2017 to increase awareness of the cash rewards for reporting guinea worm cases and infections and to publicize information about how to prevent the infection in humans and dogs. Two predecessor ministers of public health visited endemic villages in March and September 2015, respectively.

Since 2014, Chad’s GWEP, The Carter Center, and WHO have pursued a robust research agenda to help understand the unusual epidemiology of dracunculiasis in the country.^7^ Results so far appear to support the hypothesis that transmission involves a paratenic or transport host, that frogs may be more susceptible hosts than fish; that guinea worms recovered from infected dogs and from humans in Chad are indistinguishable from each other but slightly different by analysis of microsatellite and mitochondrial DNA (Liz Thiele, unpublished data) and analysis of full genome DNA (James Cotton, unpublished data) from guinea worms in other endemic countries; and that GPS collars and examination of stable isotopes in dog whiskers are feasible ways to study dog movements and dietary habits in Chad. One *D. medinensis* larva has been recovered from a wild-caught frog in Chad.^8,9^ Genetic studies also suggest that guinea worms were circulating in Chad in 2001–2009, when no cases were reported.^7^ Investigations to assess the potential effectiveness of moxidectin/imidacloprid (Advocate/Advantage^®^, Bayer) or ivermectin/pyrantel (Heartgard^®^, Merial) antihelminthic treatment as a mass prophylactic to prevent guinea worm infections in dogs in rural Chad are also underway. Additional results from these studies will be published separately.

### Ethiopia.

Ethiopia had seemed to be on the verge of success when it reported only one case in 2006 and zero cases in 2007. Ethiopia reported a total of 20 cases of dracunculiasis in humans during the 5-year period 2012–2016 before having an outbreak of 15 cases that began in September 2017. The latter outbreak occurred among migrant farm laborers from Oromia region who drank contaminated water while working at a commercial farm in adjacent Gambella Region. The program located and interviewed most laborers who worked at the commercial farm in 2016 and 2017. Although 12 of the cases in 2017 were not contained, the Ethiopia DEP (EDEP) applied Abate in areas around the farm associated with the cases. Surface sources of water in their home areas in Oromia region were all flowing streams or rivers, which is unsuitable habitat for copepods. The program began enhanced health education activities similar to those in Gambella in affected areas of Oromia in October in response to the outbreak.

The EDEP also reported a total of 34 infected domestic dogs and five infected baboons in the 5 years before 2017; in 2017, it reported 11 infected dogs (six contained) and four infected baboons. Most of the baboons were killed by dogs that were protecting crops or accompanying hunters; the guinea worms were noticed after the baboons were killed. In 2014–2016, almost all infected animals and humans with dracunculiasis as well as the infected animals in 2017 occurred in Gambella Region’s Gog district, which reported no cases in humans in 2017 for the first time in 7 years. The cases in Gog district in 2014–2016 were mostly ethnic Agnuak males older than 10 years who were hunters, gathered honey, or had other activities in or at the edge of the forest. Centers for Disease Control and Prevention confirmed worm specimens from humans, dogs, and baboons as *D. medinensis*. In 2017, a veterinarian from the GWEP at Carter Center headquarters began working with the EDEP and Ethiopian veterinary, public health and wildlife officials in preparation for a baboon–dog epidemiology and ecology project to begin in Gog district early in 2018. This will be the first time that baboons in Gambella will be the subject of such a study.

Since 2015, the EDEP has increased the numbers of surface water sources where it has applied Abate in forest areas associated with human and animal infections in Gog district 10-fold, treating 44 surface water bodies in the core endemic subdistrict of Atheti in July 2015, 131 in July 2016, and 484 in July 2017, for example, in addition to providing the villagers health education, cloth filters and pipe filters. Unlike in Chad, most of the surface water bodies of concern here are small enough to be treated with Abate but are numerous and transient. All of the five villages at high risk in Gog district have at least one safe source of drinking water. Insecurity limited activities in some affected areas of Gambella Region in December 2015–January 2016.

In 2017, Ethiopia had 167 villages under active surveillance. The EDEP increased the cash reward for reporting a case of dracunculiasis to the equivalent of US$100 in 2014 and began offering a reward equivalent to US$10 for reporting an infected animal in April 2015. In 2018, it plans to increase the rewards for reporting an infected human to US$360 and the reward for reporting and tethering infected dogs to US$40. Overall, 83% of persons interviewed in areas under active surveillance in Gambella and Southern Nations, Nationalities and People’s Regions in 2017 were aware of the cash reward for reporting a person with dracunculiasis. However, only 22% of persons interviewed in Oromia region, which had not had a case of dracunculiasis since the EDEP began and thus was not under active surveillance, were aware of the reward. Ethiopia’s minister of health visited an endemic area in Gambella Region in 2013, the vice president of Gambella Region made a similar visit in support of the EDEP in 2016, and the president of Gambella Region visited the implicated commercial farm in 2017. The National Certification Committee met for the first time in several years in 2015 but has been dormant since then. The EDEP held three press conferences to publicize the program in 2016. In December 2017, it launched an intensive communication campaign to increase awareness of the rewards nationwide. This program had three successive national coordinators in 2013–2017, which hampered program continuity.

### South Sudan.

South Sudan, which reported 521 cases of dracunculiasis in 2012, reported 70 cases in 2014 and six cases in 2016. At the end of December 2017, it reached the major milestone of 13 consecutive months with zero reported cases despite submitting 27 worm specimens to CDC during 2017, none of which was confirmed as *D. medinensis*. South Sudan’s latest case, a 13-year-old Lou girl from Jur River County in Western Bahr el Ghazal State, had her worm emerge on November 20, 2016. The South Sudan GWEP (SSGWEP) monitored all six cases from 2016 throughout 2017. It has only once detected an infected dog, and this occurred in the household of a patient with dracunculiasis in Jur River County in 2015.

South Sudan introduced a cash reward of 5,000 South Sudanese Pounds (∼US$100) for reporting a case of dracunculiasis, in April 2014, which it increased to 10,000 SSP (∼US$90) in May 2017, and 50,000 SSP (∼US$400) in January 2018. It began offering a cash reward equivalent to US$23 for reporting an infected dog in May 2017. The SSGWEP had 4,046 villages under active surveillance in 2017, and it launched a nationwide communication campaign in October 2017 to publicize the rewards and guinea worm disease prevention. A survey in Jur River and Tonj East counties in September 2017, found 72% of those queried were aware of the reward for reporting an infected person. The SSGWEP reported a provisional total of 21,116 rumors of cases in January–October 2017, of which 99% were investigated within 24 hours. The World Health Organization is helping to monitor South Sudanese refugees in Ethiopia, Uganda, and other neighboring countries for cases of the disease.

South Sudan experienced significant sporadic insecurity in some areas during the period under review, to a degree that required evacuation of 33 expatriate Carter Center–supported staff during the off-peak season in December 2013–February 2014, and again in July 2016. Only about five expatriate senior staff returned after the second evacuation, but local volunteers and indigenous supervisors continued to function at a high level under the effective leadership of the national program coordinator and continued exceptionally strong political support of the South Sudanese government. The SSGWEP’s indigenous workers included 78 administrative and transport staff, 208 supervisors, and 3,137 village volunteers in 2016, and numbered 2,306 supervisors and 18,169 village volunteers at their peak in 2007. The minister of health visited endemic villages in 2014 and 2016 and launched the nationwide communication campaign mentioned previously in 2017. In 2015, the SSGWEP’s annual review meeting in Juba was opened by the vice president of South Sudan in the presence of the governor of the highest endemic state, three national ministers, six state ministers of health, and four county commissioners.

### Mali.

Mali reported only four cases of dracunculiasis in 2012 when it also suffered a coup d’etat in March followed by virtual partition of the country that made much of the northern half of the nation inaccessible to the program for over a year, and very insecure after that. Security improved by 2014 when the national program coordinator visited Kidal region in April. Mali reported 11 cases in 2013, then had an outbreak of 40 cases in three villages in August–November 2014: 29 cases (28 contained) at Tanzikratene in Gao region, 10 cases (seven contained) at Nanguaye in Timbuktu region, and one uncontained case at Fion in Segou region. All but one of the latter cases were ethnic black Tuaregs. Neither Tanzikratene nor Nanguaye had a functioning source of safe drinking water.

Malian authorities and their partners countered the outbreak in 2014 by increasing Abate application in the three villages during the transmission season, intensifying health education, providing the inhabitants with cloth and pipe filters, and doubling the cash reward for reporting a case to the equivalent of US$100. The United Nations Children’s Fund repaired the mechanized water system in Tanzikratene in September 2015 and the German nongovernmental organization HELP, repaired it again in February 2017. The program monitored all 40 patients from 2014 throughout 2015; it had 698 villages under active surveillance and conducted additional active surveillance in cooperation with polio immunization days that year. In 2017, it still had 455 villages under active surveillance in affected areas of the country, queried more than 33,000 persons as part of polio immunization days, monitored monthly reports from health posts nationwide, and investigated 452 rumors of cases, despite ongoing insecurity. Awareness of the cash reward for reporting an infected person was estimated at 84% of persons surveyed in 2016 and 86% of persons queried in 2017. The World Health Organization helped maintain surveillance for cases in camps for internally displaced persons in Mali and among Malians in refugee camps in neighboring Burkina Faso, Mauritania, and Niger. Mali appointed a new minister of public health in 2015 and established a National Committee for Certification of Dracunculiasis Eradication in May that year. It launched a nationwide communication campaign to publicize the rewards and guinea worm disease prevention in March 2017.

After the vigorous response in 2014, Mali reported five cases (three contained) in 2015, of which three were in Tanzikratene, and one each in villages in Segou and Timbuktu regions. The final case was a 45-year-old woman in Timbuktu region whose worm emerged on November 17th. Mali reported no more persons with dracunculiasis in 2016 or 2017.

However, for the first time since the Malian program began, in October 2015 it found one dog with an emerging guinea worm in Tominian district of Segou region. The new minister of public health visited Tominian district in 2016, when Mali reported 11 infected dogs (nine contained) in that district. The program began offering a reward equivalent to US$20 for reporting and tethering any infected dog in March 2016 (78% of persons queried in 2017 were aware of that reward). It intensified health education of villagers in Tominian and a few adjacent districts where the infected dogs originated and applied Abate in surface water sources associated with the infected dogs. This area of Mali is part of the inland delta of the Niger River, with ecology somewhat similar to that along the Chari River in Chad. Mali reported nine infected dogs (eight contained) and one infected cat (contained) in 2017.

### Sudan.

In June and September 2013 three cases of dracunculiasis were discovered during a polio immunization campaign in Kafia Kinji village of Sudan’s southern Darfur region, near the borders with South Sudan and Central African Republic (CAR). All three cases were contained and all were members of the same family who became ill 11 years after the last known indigenous case in Sudan and 6 years after the last known imported case. It is believed the patients were infected by drinking water from ponds that may have been contaminated by furtive rebels in the insecure area. The Carter Center and WHO helped the Government of Sudan to establish active surveillance and implement control measures, and no further cases were discovered.^10^ An external evaluation of this program by WHO in 2016 found inadequate documentation of supervision, low awareness of the cash reward for reporting of cases, and impediments to program operations in some areas because of insecurity. Two suspected cases were reported and investigated in 2017 near or in insecure border areas with South Sudan and CAR, including active case searches in the respective immediate areas of the suspect cases, and a specimen from one of the cases was examined at CDC but neither case was confirmed. Central African Republic authorities conducted searches for cases of dracunculiasis in the areas adjoining Sudan and Chad in 2017.

### Global activities.

The global campaign held joint reviews of the four remaining country programs in February or March to review progress and discuss challenges at Ouagadougou (2013), Addis Ababa (2014), Bamako (2015), and at The Carter Center in Atlanta (2016, 2017). The ministers of health of Mali and South Sudan, the state minister of health of Ethiopia, and a representative of the minister of health of Chad participated in the 2016 review. These joint reviews, which are distinct from the in-country reviews conducted by each endemic country in December–January, also recently included the four other countries that were not yet certified by the International Commission for the Certification of Dracunculiasis Eradication (ICCDE): Angola, Democratic Republic of Congo, Kenya, and Sudan. The World Health Organization convened a second meeting of postcertified countries in Lome, Togo in 2016 (the first meeting was in 2010). The ICCDE endorsed certification of Cote d’Ivoire, Niger, and Nigeria in 2013; Ghana in 2015; and Kenya in February 2018. As of February 2018, WHO had certified 199 countries, territories, and areas as free of dracunculiasis transmission on the recommendation of the ICCDE (The ICCDE expects to consider applications of Angola, Democratic Republic of Congo, and Sudan later in 2018 or 2019.). Each May, WHO continues to host an informal meeting with ministers of health of endemic and formerly endemic countries during the WHA in Geneva. The World Health Organization director general Dr. Margaret Chan attended the informal meeting in 2014 that was even better attended than previous meetings. As required by a resolution at the WHA in 2011, WHO reports on the progress of the dracunculiasis eradication campaign to the WHA annually. Major donors to the campaign reviewed the global program with staff from The Carter Center and WHO and other experts in July 2014, as a result of which The Carter Center assumed responsibility from WHO for assisting the four remaining endemic countries to maintain surveillance in dracunculiasis-free areas and to maintain surveillance nationwide for all 3 years after the last case instead of for only 1 year. Meeting participants also revised the definition of a case of dracunculiasis to include laboratory confirmation and revised the criteria for a contained case to include use of Abate if a source of drinking water is known to have been contaminated or if there is uncertainty about contamination. The International Task Force for Disease Eradication also reviewed the global program in 2015^11^ and 2017.^7^

Former U.S. President Jimmy Carter continued his advocacy for the eradication program by writing and calling heads of state of endemic countries, meeting Sudan’s president in Khartoum in 2014, attending the inaugural launch of the exhibition “Countdown to Zero: Defeating Disease” at the American Museum of Natural History in New York City in 2015, lecturing on guinea worm eradication at Britain’s House of Lords in connection with a version of the Countdown to Zero exhibition in London in 2016, launching an adaptation of the exhibition at The Carter Center in 2016, participating in the annual review meeting that included ministers of health at The Carter Center in 2016 and a meeting with guinea worm researchers in 2017, and recording video messages to informal meetings with ministers at WHO, to a launch of the Countdown to Zero exhibition in Abu Dhabi in 2017 and to other meetings. In 2015 he also enlisted the Most Honorable Dr. World Laureate Tebebe Yemane Berhan of Ethiopia, member of the International Board of Trustees of Lions Clubs, to become a goodwill ambassador for guinea worm eradication in the four remaining endemic countries.

During 2013–2017, the WHO Collaborating Center for Dracunculiasis Eradication at the CDC examined 861 specimens thought to be guinea worms submitted by countries. It confirmed 275 of them as *D. medinensis* by microscopic and/or molecular evaluation.^3^ One measure of the increasing intensity of surveillance has been recognition of an unprecedented number of spargana infections in humans in Ethiopia and South Sudan.^12^ In association with The Carter Center, the Collaborating Center at the CDC continues to issue the newsletter of the global campaign, *Guinea Worm Wrap-Up*, almost monthly. Summaries of the status of the campaign are published annually in the CDC’s *Morbidity and Mortality Weekly Report*^13^ and in WHO’s *Weekly Epidemiological Record*.^14^

## DISCUSSION

The world is now closer than ever to eradicating dracunculiasis, with cases in humans reported in only Chad and Ethiopia in 2017. No cases were reported worldwide for the first time in January 2015 and in five other months in 2015–2017. In theory, the last person to suffer from this disease could occur at any time, but we will not know when we will have reached that milestone until no cases are detected for a year or more after it happens.

At our previous review in this journal 5 years ago,^1^ we did not and would not have predicted that Mali and South Sudan, the two countries with the most insecurity then and now, would become the first two of the final four to interrupt transmission to humans. Surveillance is imperfect in both countries because of sporadic insecurity in some areas but daily acts of courage by dedicated nationals and some expatriates have produced substantial relevant documentation nonetheless. The program of South Sudan enjoyed exceptional political support from the country’s leaders and the technical ability of its national coordinator, which helped it overcome challenges of complex epidemiology and weak infrastructure as well as ongoing insecurity. Mali has had no known cases in humans in 2016 or 2017, despite detecting 20 infected dogs and a cat in a small partly insecure area of the country in those 2 years after transmission to humans was interrupted.

Apart from insecurity and the challenges of maintaining sensitive surveillance in the areas of concern, the biggest challenge to completing eradication now is the large number of infected dogs in Chad, where it appears that the numerous infections in dogs are driving the sporadic infections with the same *D. medinensis* in humans. Throughout the global campaign previously, and in a few other observed reports in the former Soviet Union^15,16^ and elsewhere earlier, transmission among humans appeared to be the fundamental driving force of guinea worm infections, with incidental infection of dogs. The reverse appears to be true in Chad, where dog infections appear to be the main driving force of infection, with incidental infection of humans. We assume that the few guinea worm infections in dogs that have been reported in some formerly endemic South Asian countries after 1950 and that were called *D. medinensis* but not analyzed genetically, were probably due to an unknown zoonotic *Dracunculus* species, because they were not associated with any known human infections. With few infected dogs, Mali and Ethiopia will soon show whether past experience that predicts the disappearance of *D. medinensis* infections in dogs after human cases cease will be realized there. An additional unknown being researched in Chad is the potential role of paratenic transmission via an amphibian host. In Ethiopia the few known infections in baboons are also being researched but current indications are that infected baboons are handicapped and hence more likely to be killed by dogs and that the small numbers of dogs, baboons, and residual endemic human cases are associated with forest activities in a very small area where assiduous application of Abate should stop transmission to all.

As noted previously, it now appears that transmission of infections with guinea worms continued in Chad during the decade when no cases were reported in humans and before infections were discovered in domestic dogs there. Why the outbreak among dogs appeared in Chad when it did and the reason for the paradoxical and unique occurrence in Chad of *D. medinensis* so commonly in dogs but so infrequently in humans may never be answered. Ecological changes associated with over-fishing and drought may have played a role, but elders and leaders of the earlier campaign confirm that infections in dogs were not seen before the current outbreak and that seasonal mass fishing has been practiced along the river for generations. There is no evidence that the slight genetic difference in guinea worms in Chad has a significant role in the epidemiological peculiarity of the current outbreak there. The first reductions in dog infections in 2017, the continued intensification of available interventions, and the robust research agenda in Chad are reasons to expect that this final challenge also will be overcome soon.
